# Electrical Conductivity of Ionic Liquids 1-Hexyl-3-Methylimidazolium Chloride (HMIM) and 1-Methyl-3-Octylimidazolium Chloride (OMIM) in Mixtures with Selected Alkoxy Alcohols over a Wide Temperature Range

**DOI:** 10.3390/molecules28237831

**Published:** 2023-11-28

**Authors:** Zdzisław Kinart

**Affiliations:** Department of Physical Chemistry, Faculty of Chemistry, University of Lodz, Pomorska 163/165, 90–236 Lodz, Poland; zdzislaw.kinart@chemia.uni.lodz.pl

**Keywords:** electric conductivities, alkoxy alcohols, ionic liquids, thermodynamic function

## Abstract

Ionic liquids have been the subject of intense research because of their unique electrochemical properties and potential applications in various fields. In this article, we analyze the electrical conductivity of two selected ionic liquids, 1-hexyl-3-methylimidazolium chloride (HMIM) and 1-eethyl-3-octylimidazolium chloride (OMIM), in various alkoxy alcohols such as 2-methoxyethanol, 2-ethoxyethanol, 2-propoxyethanol and 2-butoxyethanol. Our research focuses on attempting to analyze the impact of the molecular structure of both the ionic liquids and alkoxy alcohols on their electrical conductivity properties. The results of our study can be highly beneficial in the design of advanced electrochemical materials and their various applications.

## 1. Introduction

In today’s world, physical chemistry is a scientific field that shapes our understanding of fundamental chemical processes and provides essential tools for designing new materials and compounds with unique properties [1,2,3]. An area that plays a significant role in this field is the study of ionic liquids, which comprise a fascinating group of substances with distinctive physical and chemical properties. Ionic liquids, also known as liquid salts, consist of cations (organic) and anions (organic or inorganic), forming a liquid phase with exceptional electrochemical and thermodynamic properties.

Thanks to the properties mentioned above, ionic liquids already have and can have even more applications. They can replace flammable and unstable organic solvents used in chemical processes [1,2]. They are used as absorbents in gas separation and as working fluids (lubricants, heat transfer fluids, hydraulic fluids) [4]. A notable example is the ethylsulfate 1-ethyl-3-methylimidazolium, which has been used as a hydraulic fluid [5] and is known under the commercial name ECOENGTM 212. Furthermore, ionic liquids are used in extraction processes [6,7], biofuel production and biobutanol extraction [8,9]. More frequently, the hexafluorophosphate 1-butyl-3-methylimidazolium [C4C1im] [PF6] is used as an extractant, as it has properties similar to 1-octanol, it extracts benzene, salicylic acid, and aniline from water [10]. Ionic liquids can be used for nuclear fuel production [11]. At first, ionic liquids in the form of chloroglinian salts were employed by electrochemists, utilizing them as electrolytes for high-energy-density cells. This translates into the use of ionic liquids in lithium batteries [12,13]. They are also used in baths for the electrolytic deposition of metals and their alloys. Ionic liquids are good lubricants, bactericidal and fungicidal agents, tissue fixatives and balms, antistatic agents, surfactants, ink components, have the ability to dissolve cellulose, are selective adsorbents for sulphur compounds from gasoline and oils [14,15,16,17,18]. However, ionic liquids are primarily used in organic synthesis, including Friedel–Crafts reactions, Diels–Alder reactions, dimerization, oligomerization, olefin polymerization, depolymerization, cyclopropanation, halogenation, nitration, oxidation, epoxidation, isomerization, catalytic hydrogenation, esterification, Heck, Suzuki–Miyaura reactions [19,20,21,22,23,24,25]. The use of ionic liquids ensures high reaction efficiency and increases chemo, regio, stereo, and enantioselectivity. In addition to selective separation of reaction products, it is also easier to separate them from the catalyst. Ionic liquids are also used in biocatalytic processes, where they can act as a pure solvent, a solvent mixture, or a separate phase, and most importantly, they do not deactivate the enzymes or cells used or do so to a very small extent [26,27,28]. It is also worth mentioning the first successful industrial application of ionic liquids, which changed the attitude of many sceptics that “ionic liquids will never be used in industry” [29].

What makes ionic liquids even more intriguing is their ability to form complexes and interact with other chemical compounds (particularly alkoxy alcohols). Alkoxy alcohols are chemical compounds that contain both a hydroxyl group (-OH) and an alkoxy group (-RO) in one molecule. This unique structure gives alkoxy alcohols the ability to interact strongly with various types of ions and particles in ionic liquids.

Understanding the mechanisms of interaction between alkoxy alcohols and ionic liquids is not only a scientific challenge but also of great significance in the context of potential practical applications of these systems. The electrical conductivity of ionic liquids is a key property of these substances, enabling their use in various electrochemical and biomedical processes.

In recent years, research interest in ionic liquids has shifted towards the analysis of their behaviour in the presence of other chemical substances, particularly alkoxy alcohols. Alkoxy alcohols, such as 2-Methoxyethanol, 2-Ethoxyethanol, 2-Propoxyethanol, and 2-Butoxyethanol, are important components of many industrial processes and chemical products [30,31,32,33,34,35]. Their ability to form complexes with ionic liquids can significantly influence their properties, opening new possibilities for the use of these substances.

In this article, we present the results of our research on the electrical conductivity of two ionic liquids: 1-Hexyl-3-Methylimidazolium Chloride (HMIM) and 1-Methyl-3-Octylimidazolium Chloride (OMIM) in the presence of selected alkoxy alcohols. Our goal is to understand how the molecular structure of the ionic liquids and alkoxy alcohols affects their electrical conductivity. The aim of this paper is to answer questions regarding the mechanisms of intermolecular interactions and the role of functional groups in these processes.

## 2. Results and Discussion

The conductivity data were analyzed using the Fuoss-Justice equation [36,37] according to the low concentration Chemical Model (lcCM) [38] This approach utilizes the following set of equations:

(1) $$\Lambda_{m}=\alpha [\Lambda_{m}^{0}-S(\alpha c{)}^{\frac{1}{2}}+E\left(\alpha c\right)+J\left(\alpha c\right)-J_{\frac{3}{2}}(\alpha c{)}^{\frac{3}{2}}]$$

together with

(2) $$K_{A}=\frac{1-\alpha }{\alpha^{2}cy_{\pm }^{2}}$$

and

(3) $$\ln y_{\pm }=-\frac{A\alpha^{1/2}c^{1/2}}{1+Br\alpha^{1/2}c^{1/2}}$$

In these equations, $\Lambda_{m}^{0}$ is the limiting molar conductivity; α is the dissociation degree of an electrolyte; *K_A_* is the ionic association constant; *R* is the distance parameter of ions; y_±_ is the activity coefficient of ions on the molar scale; A and B are the Debye–Hückel equation coefficients. Calculations were carried out assuming that *R = q* (*q*—Bjerrum distance [39]

The analytical form of the parameters S, E, J and J^3/2^ has previously been presented [38,40] The calculation was performed as described by Bešter-Rogač [41,42].

In the present study, the electric conductivities for the ILS were measured in the temperature range 283.15–313.15 K (in 5 K steps), thus allowing the limiting molar conductivity and the association constants of the examined ionic liquids to be estimated. 

An analysis of the electrical conductivity of two ionic liquids was performed, 1-hexyl-3-methylimidazolium chloride (HMIM) and 1-methyl-3-octylimidazolium chloride (OMIM), at different concentrations of alkoxy alcohols: 2-methoxyethanol, 2-ethoxyethanol, 2-propoxyethanol and 2-butoxyethanol was conducted. Analysis of the physicochemical parameters for pure solvents is presented in Table S1 [43,44,45,46,47,48,49,50,51]. These values were compared to the values of the literature, which are essential for optimizing the obtained data. The results of electrical conductivity show that the electrical conductivity of ionic liquids increases with an increase in the concentration of alkoxy alcohols. This phenomenon is consistent with what has been reported in the literature, suggesting that the presence of other molecules in the ionic liquid can increase the mobility and improve conductivity.

In Table S2, all molar conductivity values and concentrations are compiled as a functions of temperature. These values also exhibit a linear relationship. The research results demonstrated that the electrical conductivity of HMIM and OMIM is significantly dependent on the type and concentration of alkoxy alcohols. Significant differences in electrical conductivity were observed between the alkoxy alcohols, indicating the crucial role of their chemical structure in this process.

**Figure 1:**
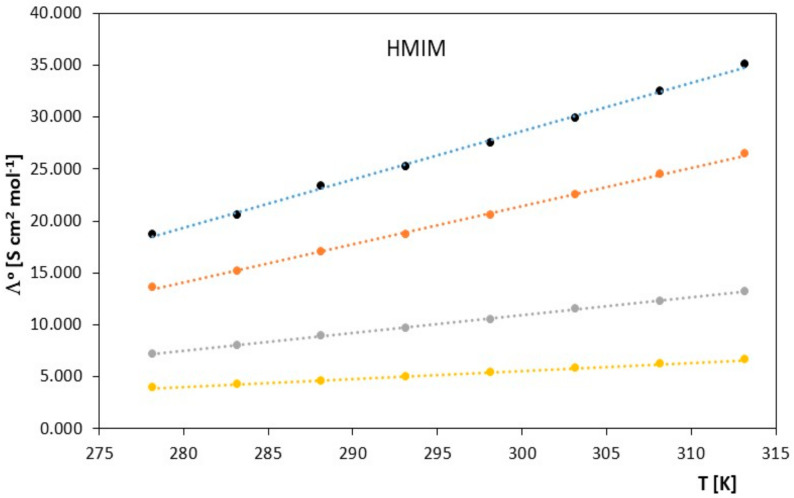
The figures depict the relationship of the limiting molar conductivity Λ*^o^* [S·cm^2^·mol^−1^] as a function of temperature *T* [K] for the studied ionic liquid, 1-hexyl-3-methylimidazolium chloride [HMIM] in the presence of alkoxy alcohols (● 2-methoxyethanol, ● 2-ethoxyethanol, ● 2-propoxyethanol, ● 2-butoxyethanol).

**Figure 2:**
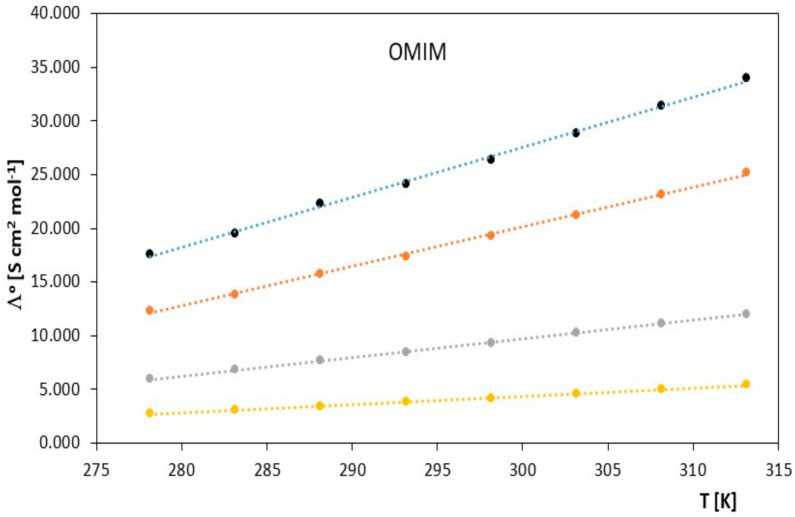
The figures depict the relationship of the limiting molar conductivity Λ*^o^* [S·cm^2^·mol^−1^] as a function of temperature *T* [K] for the studied ionic liquid, 1-methyl-3-octylimidazolium chloride [OMIM], in the presence of alkoxy alcohols (● 2-methoxyethanol, ● 2-ethoxyethanol, ● 2-propoxyethanol, ● 2-butoxyethanol).

Limiting molar conductivity values increase with increasing temperature. The physicochemical properties of the solvent also have a significant impact on the molar conductivity values. From the data collected in Table 1 and presented in Figure 1 and Figure 2, it can be concluded that at a given temperature, the limiting molar conductivity values of the studied ionic liquids (in the alkoxy alcohols) decrease with increasing molar mass of the studied alkoxy alcohols. Such changes in limiting molar conductivity were expected, and it can be assumed that the main factor influencing the values of ionic association is the relative permittivity of the solvent. As in the case of classical electrolytes, low solvent permeability favors strong Coulombic attraction and the formation of stable ion pairs.

The discussed values in alkoxy alcohols have a linear character in accordance with Fuoss’s assumptions [40]. The molar conductivity of both ionic liquids discussed in the article takes the opposite order compared to association constants. The molar conductivity values are closely related to viscosity. Very interesting conclusions can be drawn that when we increase the molar mass of the examined ionic liquid (i.e., its size), the value of the association constant increases.

**Figure 3:**
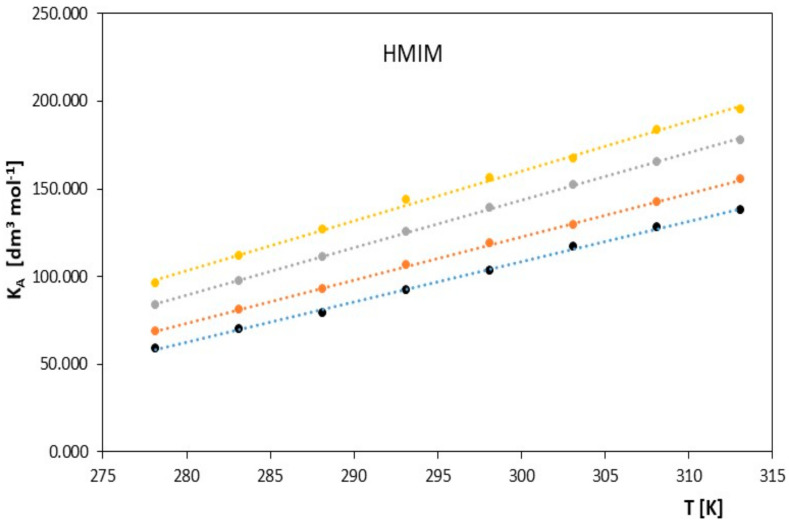
The plots of the association constant, *K_A_* [dm^3^·mol^−1^], as a function of temperature, *T* [K], for the investigated ionic liquid, 1-hexyl-3-methylimidazolium chloride [HMIM] with alkoxy alcohols (● 2-methoxyethanol, ● 2-ethoxyethanol, ● 2-propoxyethanol, ● 2-butoxyethanol.

**Figure 4:**
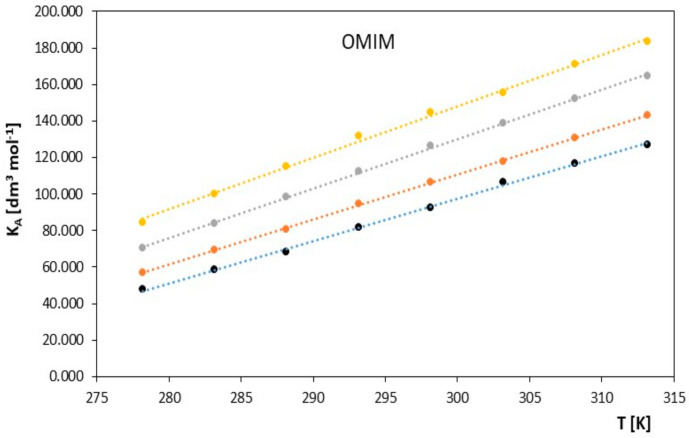
The plots of the association constant, *K_A_* [dm^3^·mol^−1^], as a function of temperature, *T* [K], for the investigated ionic liquid 1-methyl-3-octylimidazolium chloride [OMIM], with alkoxy alcohols (● 2-methoxyethanol, ● 2-ethoxyethanol, ● 2-propoxyethanol, ● 2-butoxyethanol.

In contrast, the values of the association constant *K_A_* for the studied ionic liquids change with temperature, as is evident in Table 1. These values increase with increasing temperature for the studied ionic liquid and with the increasing molar mass of the solvent: METHOXY < ETHOXY < PROPOXY < BUTOXY. However, this trend reverses with increasing molar mass of the ionic liquid. OMIM < HMIM, which is clearly visible in Figure 3 and Figure 4.

It can be assumed that the mobility of ions in infinite dilution is mainly controlled by factors related to macroscopic viscosity, and weak ion solvation is expected in these systems. A detailed analysis of solvation processes based on changes in the limiting ionic conductivities and Stokes radii in alkoxy alcohols indicates that the investigated ionic liquids are weakly solvated by solvent molecules. Ion–solvent interactions for the Cl^−^ anion are also weak but slightly stronger than for large organic cations in ionic liquids.

The electrical conductivity studies of HMIM and OMIM in the presence of alkoxy alcohols using the conductometric method provided significant insight into these chemical systems. The influence of alkoxy alcohol concentration on the conductivity showed that the electrical conductivity of HMIM and OMIM increases with increasing alkoxy alcohol concentration. This is an expected phenomenon because a higher concentration of alkoxy alcohols introduces more charged particles into the solution, thus increasing the ability to conduct an electric current. The differences between types of alkoxy alcohols inform us that variations in their structure have significant effects on the influence of different alkoxy alcohols on the ionic liquid conductivity. Some alkoxy alcohols, such as 2-methylethanol, can impact conductivity more efficiently than others. This suggests that the molecular structure of alkoxy alcohols plays a crucial role in the interactions between them and ionic liquids.

Analyzing our results, we observed that different alkoxy alcohols affect the electrical conductivity of ionic liquids differently. For example, 2-methoxyethanol appears to be more effective in increasing conductivity than 2-propoxyethanol. This suggests that the molecular structure of alkoxy alcohols, including the length of the alkoxyl chain, is essential to the intermolecular interactions between alkoxy alcohol and the ionic liquid. These intermolecular interactions confirm the presence of strong intermolecular interactions between alkoxy alcohols and ionic liquids. These intermolecular interactions affect the ability to conduct an electric current through the solution. Understanding the impact of alkoxy alcohols on the conductivity of ionic liquids has potential applications in various fields, such as electrochemistry, the chemical industry, and materials science. This could lead to the design of advanced electrochemical materials with controlled conductivity properties.

These conclusions serve as a starting point for further research in the field of electrical conductivity of ionic liquids and their interactions with various chemical compounds. These studies may lead to the discovery of more detailed mechanisms of intermolecular interactions and more precise control of conductivity in these systems.

In summary, the results obtained indicate that the conductometric method is an invaluable tool in the study of electrical conductivity of HMIM and OMIM in the presence of alkoxy alcohols. It provides essential information about the influence of molecular structure and concentration on the electrochemical properties of these systems, which has the potential to create new materials and applications in industry and science. Electrical conductivity studies allowed for a thermodynamic analysis of the process, providing us with tools to understand the changes that occur in these systems and the forces driving these changes. The results are presented in Table 2 and Table 3.

The temperature dependence of the association constant was used to calculate the Gibbs free energy, Δ*G*^0^:

Δ*G*^0^*(T)* = −*R T lnK*_*A*_*(T)* (4)

Δ*G*^0^*(T)* can also be expressed by the polynomial equation:

Δ*G*^0^*(T)* = *A* + *B T* + *C T*^2^ (5)

The entropy, Δ*S*^o^, and enthalpy, Δ*H*^0^, of ion association are defined as:

(6) $$\Delta S^{0}\left(T\right)=-(\frac{\delta \Delta G^{0}}{\delta T}{)}_{p}=-B-2CT$$

Δ*H*^0^
*=* Δ*G^o^ + T* Δ*S*^0^ (7)

**Figure 5:**
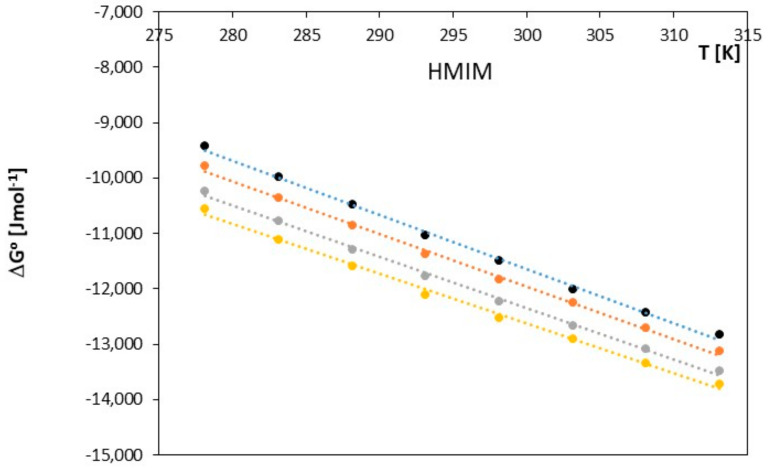
The figures shows the dependence of Δ*G^0^* [J∙mol^−1^] as a function of *T* [K] for ionic liquid, 1-hexyl-3-methylimidazolium chloride [HMIM], with alkoxy alcohols (● 2-methoxyethanol, ● 2-ethoxyethanol, ● 2-propoxyethanol, ● 2-butoxyethanol).

**Figure 6:**
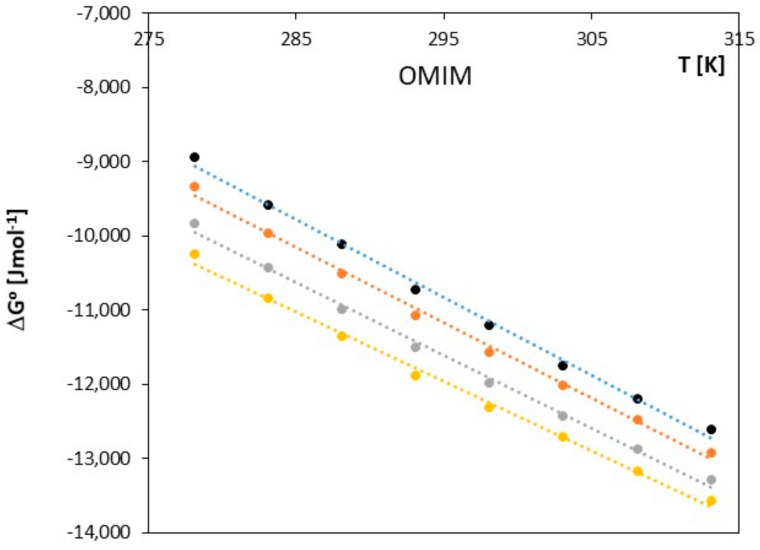
The figures shows the dependence of Δ*G^0^* [J∙mol^−1^] as a function of *T* [K] for ionic liquid 1-methyl-3-octylimidazolium chloride [OMIM], with alkoxy alcohols (● 2-methoxyethanol, ● 2-ethoxyethanol, ● 2-propoxyethanol, ● 2-butoxyethanol).

**Figure 7:**
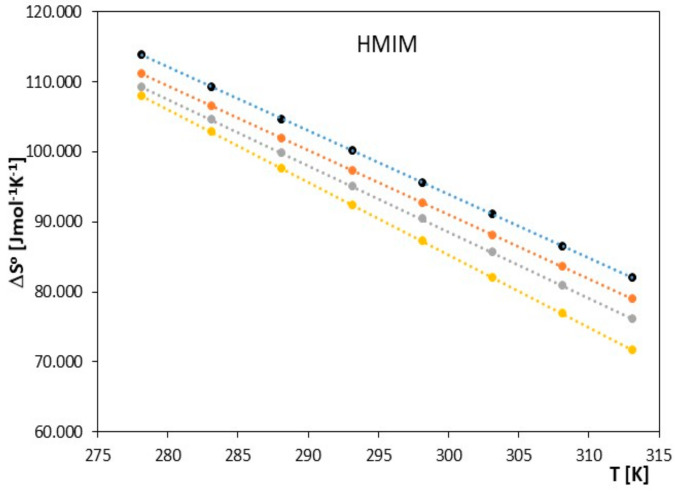
The graph depicts the relationship between Δ*S*^0^ [J∙mol^−1^·K^−1^] and *T* [K] for ionic liquid, 1-hexyl-3-methylimidazolium chloride [HMIM], with alkoxy alcohols (● 2-methoxyethanol, ● 2-ethoxyethanol, ● 2-propoxyethanol, ● 2-butoxyethanol).

**Figure 8:**
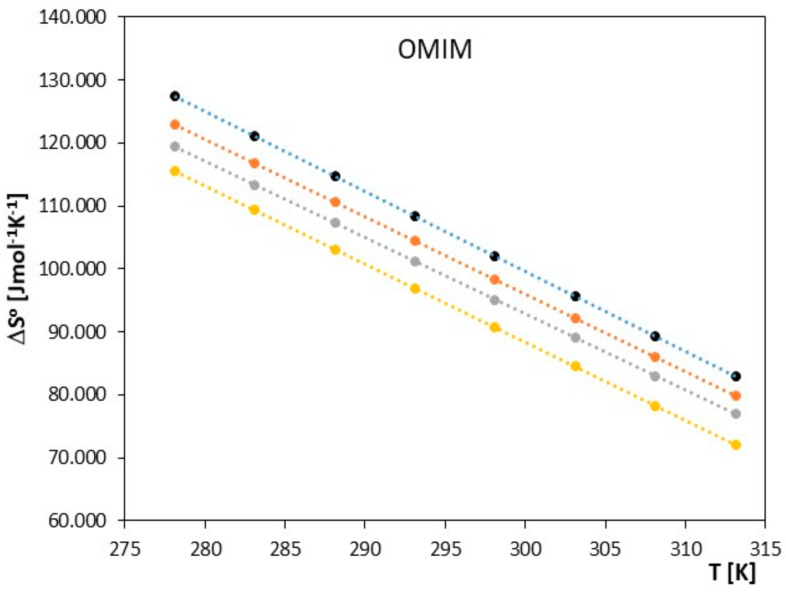
The graph depicts the relationship between Δ*S*^0^ [J∙mol^−1^·K^−1^] and *T* [K] for ionic liquid, 1-methyl-3-octylimidazolium chloride [OMIM], with alkoxy alcohols (● 2-methoxyethanol, ● 2-ethoxyethanol, ● 2-propoxyethanol, ● 2-butoxyethanol).

**Figure 9:**
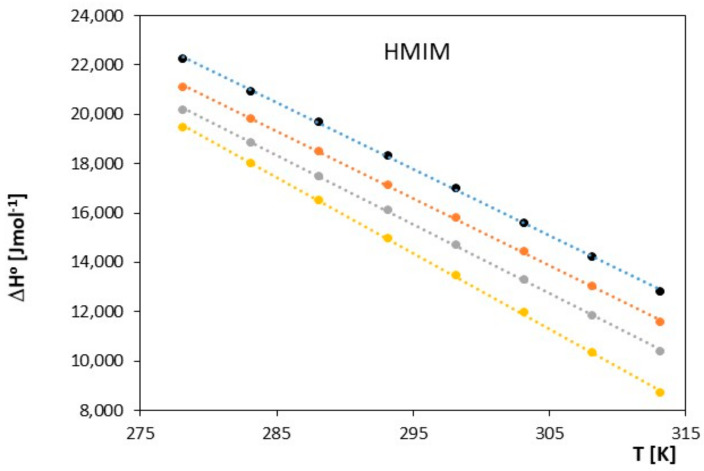
The graph illustrates the relationship between Δ*H*^0^ [J∙mol^−1^] and *T* [K] for ionic liquid, 1-hexyl-3-methylimidazolium chloride (HMIM) with alkoxy alcohols (● 2-methoxyethanol, ● 2-ethoxyethanol, ● 2-propoxyethanol, ● 2-butoxyethanol).

**Figure 10:**
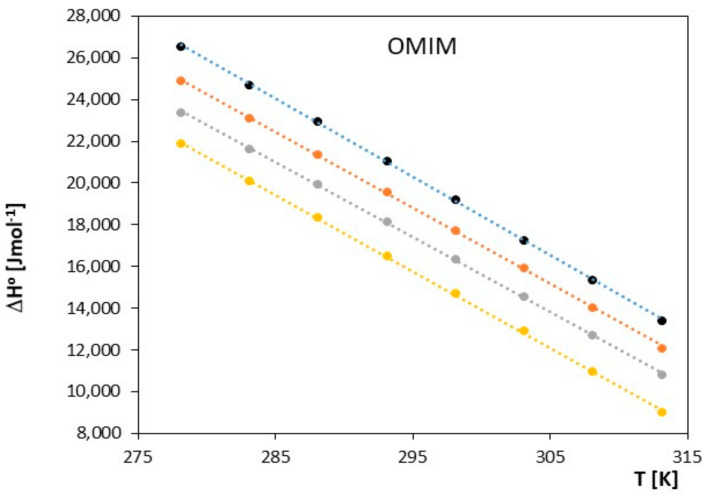
The graph illustrates the relationship between Δ*H*^0^ [J∙mol^−1^] and *T* [K] for ionic liquid, 1-methyl-3-octylimidazolium chloride [OMIM], with alkoxy alcohols (● 2-methoxyethanol, ● 2-ethoxyethanol, ● 2-propoxyethanol, ● 2-butoxyethanol).

The obtained results clearly indicate that in the examined systems, the process of ion pair formation is spontaneous, and this effect increases with temperature. Most likely, an increase in temperature leads to a reduction in the interaction between the ionic liquid and the investigated alkoxy alcohol, resulting in a decrease in the preferential solvation of ions with an increase in temperature. In our studies, we also observed that as the cation radius decreases in the examined ionic liquids, there is a slight decrease in the spontaneity of ion pair formation. As shown in Figure 5, Figure 6, Figure 7, Figure 8, Figure 9 and Figure 10, both entropy and enthalpy values are positive. Additionally, the values of Δ*S*^0^ and Δ*H*^0^ decrease with increasing temperature. The trends in both functions indicate that the transition from freely solvated ions to ion pairs makes the system less ordered (this conclusion applies to the positive entropy values describing the analyzed process). However, the observed positive values of Δ*H*^0^ suggest that the process of ion pair formation is endothermic (see Table 3). The analysis of thermodynamic functions describing the associative processes investigated in this study indicates that for alkoxy alcohols, the values of Δ*H*^0^ and Δ*S*^0^ are positive over the entire temperature range. This suggests the dominant role of entropy in the ion pair formation processes in the studied systems. It should be assumed that the mere attraction of anions and cations through Coulombic forces and van der Waals forces, as well as weak hydrogen bonds, may not be sufficient to promote ion pair formation. For the examined series of ionic liquids, it seems that enthalpy values mainly depend on the nature of the anion rather than the cation. Analyzing the obtained results, we can conclude that Coulombic attraction between the anion and the cation may not be the sole factor influencing Δ*H*^0^ values, as evident in Table 3.

Changes in the association constant with temperature were used to calculate the Gibbs free energy for this process according to Equation (5). The enthalpy and entropy of the association are defined using the relationships described by Equations (6) and (7). The values of the studied thermodynamic functions *(*∆*G*^0^, ∆*S*^0^, ∆*H*^0^) in the discussed temperature range of 278.15 K–313.15 K are presented in Table 2 for 1-hexyl-3-methylimidazolium chloride (HMIM) and in Figure 5 and Figure 6.

The Gibbs energy for 1-methyl-3-octylimidazolium chloride [OMIM] strongly correlates with the temperature of the investigated process. As the temperature begins to rise, we observe that Δ*G^o^* becomes more negative. This is most likely associated with the fact that the interactions between 1-methyl-3-octylimidazolium chloride and alkoxyl alcohols become weaker at higher temperatures, as depicted in Graphs 5–6 and Table 2. The ion pairs formed are more likely to occur as the temperature of the process increases. In both studied ionic liquids, the formation of ion pairs occurs at a similar rate, and no significant differences are observed.

As seen in Figure 7, Figure 8, Figure 9 and Figure 10, both the association entropy and enthalpy values are positive, and the same is true for the second ionic liquid, 1-methyl-3-octylimidazolium (see Figure 9 and Figure 10).

The values of thermodynamic functions are significantly dependent on the molar mass of the investigated ionic liquids, as evident in the presented graphs. As the molar mass of the studied ionic liquid increases, the degree of ion association becomes progressively smaller. Stepwise processes are positive, leading to the ion pairs’ formation having a tendency towards an endothermic process.

On the other hand, the Gibbs free energy is negative, indicating that entropic processes will tend to dominate over enthalpic ones.

It is worth noting that the presence of alkoxy alcohols introduces additional thermodynamic variables into the system. We can analyze how the concentration of alkoxy alcohols affects the thermodynamics of electrical conductivity. For example, an increase in the concentration of alkoxy alcohol can increase Δ*G*^0^ of the process by influencing Δ*H*^0^ and Δ*S*^0^.

The presented thermodynamic values suggest the spontaneity of the association process, as Δ*G*^0^ < 0, making the formation of ion pairs a spontaneous process. Similarly to all of the solvents discussed above, the spontaneity of the ion association process is primarily due to changes in entropy and the ion pair formation process.

In summary, the thermodynamic analysis of electrical conductivity in HMIM and OMIM in the presence of alkoxy alcohols can provide a deeper understanding of the forces and energy changes that affect the ability of these systems to conduct electrical current. These studies can help identify key factors that influence the thermodynamics of the process and can be useful in designing electrochemical materials with specific conductivity properties.

## 3. Materials and Methods

### 3.1. Materials

For conductometric measurements, two ionic liquids were used: 1-hexyl-3-methylimidazolium chloride and 1-methyl-3-octylimidazolium chloride. Four alkoxy alcohols were used as solvents: 2-methoxyethanol, 2-ethoxyethanol, 2-propoxyethanol and 2-butoxyethanol. All reagents used were of high purity. All the necessary details are provided in Table 4.

### 3.2. Conductometric Measurements

The experimental procedure for conductometric measurements is detailed in the references [52,53,54]. Electrical conductivity measurements were conducted using an RLC Wayne-Kerr 6430B conductometric bridge with an uncertainty of 0.02%. A three-electrode conductivity cell made of non-sodium glass was used, as described in [55]. The calibration of the measuring cell was performed using an aqueous solution of potassium chloride with a sample purity of 0.99999 (Merck) [56]. Temperature control was achieved with a BU 20F calibration thermostat (Lauda, Germany) with stability better than 0.005 K, and the temperature was measured using an Amarell 3000TH AD thermometer (Amarell, Germany). The thermostat was connected to a DLK 25 flow cooler (Lauda, Germany).

Electrical conductivity measurements were conducted at various frequency values: *v* = 0.2, 0.5, 1.0, 1.5, 2.0, 3.0, 5.0, 10.0, and 20.0 kHz. Standard uncertainties are provided in Tables S2 and S3. The estimated error, composed of several factors such as the calibration of the measuring vessel, and the purity of the sample, was ±0.05%. The complete description of the electrical conductivity measurement procedure is available in reference [36].

All solutions were prepared gravimetrically using an analytical balance (Sartorius RC 210D) with an accuracy of ±1∙10^−5^ g and analyzed according to a similar procedure proposed in the works of Bešter-Rogač [57,58].

In the study, measurements of the conductivity of ionic liquids were conducted in the temperature range of 278.15 to 313.15 K (with a 5 K interval), allowing for the calculation of limiting molar conductivities, association constants, and thermodynamic functions in the investigated alkoxy alcohols.

The density of pure solvents was measured with a fully automated DSA 5000 Anton Paar (Austria) apparatus with a measurement accuracy of ±5∙10^−6^ g∙cm^−3^ and repeatability of ±10^−6^ g∙cm^−3^. The thermal control and stability over the entire investigated temperature range is estimated to be more than 0.01 K.

## 4. Conclusions

In this article, we discussed the results of our research on the electrical conductivity of HMIM and OMIM in the presence of various alkoxy alcohols. 

The molar conductivity of diluted solutions of 1-hexyl-3-methylimidazolium chloride and 1-methyl-3-octylimidazolium chloride in alkoxy alcohols was presented for the first time in the literature. Conductivity data were analyzed using Barthel’s low concentration Chemical Model (lcCM). The investigated ionic liquids in the analyzed temperature range behave as weak electrolytes, and the tendency to form ion pairs increases with temperature. The values of Δ*H*° are positive, suggesting that the ion evaporation process is endothermic. Since Gibbs free energy is negative, entropic effects seem to dominate over enthalpic effects.

The solubility decreases with increasing temperature due to the weakening of ion interactions with the solvent. This suggests that with increasing molar mass of the ionic liquid, the intermolecular interactions weaken and the number of intermolecular associations decreases.

Our work emphasizes the importance of the molecular structure of both ionic liquids and alkoxy alcohols in the regulation of their electrical conductivity properties.

On the basis of our findings and the existing literature, it can be concluded that the electrical conductivity of HMIM and OMIM ionic liquids is significantly modified by the presence of alkoxy alcohols. The differences in conductivity between various alkoxy alcohols highlight the crucial role of the molecular structure of these substances in regulating the electrical conductivity properties. Intermolecular interactions such as hydrogen-bonding and hydrophobic interactions play a key role in the formation of complexes between alkoxy alcohols and ionic liquids, affecting their conductivity.

## Figures and Tables

**Table 1:** Values of limiting molar conductivity *Λ*^o^ [S·cm^2^·mol^−1^] and association constant *K_A_* [dm^3^·mol^−1^] for the ionic liquid 1-hexyl-3-methylimidazolium chloride (HMIM) and 1-methyl-3-octylimidazolium chloride (OMIM) with alkoxy alcohols (2-methoxyethanol, 2-ethoxyethanol, 2-propoxyethanol, 2-butoxyethanol).

*T*/K	*Λ*^o^ [S·cm^2^·mol^−1^]	*K*_A_ [dm^3^·mol^−1^]	*σ* (*Λ*^o^) [S·cm^2^·mol^−1^]	*Λ*^o^ [S·cm^2^·mol^−1^]	*K*_A_ [dm^3^·mol^−1^]	*σ* (*Λ*^o^) [S·cm^2^·mol^−1^]
1-Hexyl-3-Methylimidazolium Chloride [HMIM] + 2-Methoxyethanol				1-Methyl-3-Octylimidazolium Chloride [OMIM] + 2-Methoxyethanol		
278.15	18.71	58.87	0.01	17.61	47.85	0.01
283.15	20.60	69.71	0.01	19.50	58.65	0.02
288.15	23.42	79.35	0.01	22.32	68.32	0.01
293.15	25.21	92.49	0.02	24.11	81.52	0.01
298.15	27.50	103.38	0.02	26.40	92.40	0.02
303.15	29.93	117.35	0.02	28.83	106.32	0.02
308.15	32.51	127.89	0.02	31.41	116.91	0.03
313.15	35.09	138.18	0.03	33.99	127.15	0.02
1-hexyl-3-methylimidazolium chloride [HMIM] + 2-ethoxyethanol				1-methyl-3-octylimidazolium chloride [OMIM] + 2-ethoxyethanol		
278.15	13.66	68.82	0.01	12.36	56.85	0.01
283.15	15.17	81.22	0.02	13.87	69.25	0.02
288.15	17.02	92.91	0.01	15.72	80.95	0.01
293.15	18.72	106.42	0.01	17.42	94.45	0.01
298.15	20.61	118.72	0.01	19.30	106.74	0.02
303.15	22.51	129.61	0.02	21.21	117.65	0.03
308.15	24.51	142.49	0.01	23.20	130.52	0.01
313.15	26.51	155.29	0.02	25.21	143.32	0.01
1-hexyl-3-methylimidazolium chloride [HMIM] + 2-propoxyethanol				1-methyl-3-octylimidazolium chloride [OMIM] + 2-propoxyethanol		
278.15	7.18	83.55	0.02	5.98	70.52	0.03
283.15	8.01	97.18	0.01	6.81	84.15	0.02
288.15	8.91	111.28	0.01	7.71	98.25	0.01
293.15	9.65	125.35	0.02	8.45	112.32	0.02
298.15	10.54	139.34	0.02	9.34	126.31	0.01
303.15	11.51	152.15	0.02	10.31	139.12	0.02
308.15	12.32	165.22	0.01	11.12	152.19	0.01
313.15	13.22	178.02	0.02	12.02	164.90	0.01
1-hexyl-3-methylimidazolium chloride [HMIM] + 2-butoxyethanol				1-methyl-3-octylimidazolium chloride [OMIM] + 2-butoxyethanol		
278.15	3.95	96.29	0.01	2.75	84.32	0.02
283.15	4.31	112.12	0.01	3.11	100.15	0.01
288.15	4.58	126.95	0.01	3.38	114.98	0.02
293.15	5.01	143.84	0.02	3.81	131.85	0.01
298.15	5.39	156.48	0.01	4.19	144.52	0.02
303.15	5.81	167.58	0.01	4.61	155.62	0.02
308.15	6.21	183.39	0.01	5.01	171.42	0.02
313.15	6.63	195.38	0.01	5.43	183.42	0.02

**Table 2:** Values of ∆*G^0^* [J∙mol^−1^], for ionic liquids 1-hexyl-3-methylimidazolium chloride [HMIM] and 1-methyl-3-octylimidazolium chloride [OMIM] with alkoxy alcohols (2-methoxyethanol, 2-ethoxyethanol, 2-propoxyethanol, 2-butoxyethanol) as a function of temperature.

Δ*G^0^*/[J·mol^−1^]				
1-Hexyl-3-Methylimidazolium Chloride [HMIM]				
T [K]	2-Methoxyethanol	2-Ethoxyethanol	2-Propoxyethanol	2-Butoxyethanol
278.15	−9424	−9785	−10,234	−10,562
283.15	−9991	−10,351	−10,774	−11,110
288.15	−10,478	−10,856	−11,289	−11,604
293.15	−11,034	−11,376	−11,775	−12,110
298.15	−11,498	−11,841	−12,238	−12,525
303.15	−12,010	−12,261	−12,665	−12,908
308.15	−12,429	−12,705	−13,085	−13,352
313.15	−12,832	−13,136	−13,491	−13,733
1-methyl-3-octylimidazolium chloride [OMIM]				
T [K]	2-methoxyethanol	2-ethoxyethanol	2-propoxyethanol	2-butoxyethanol
278.15	−8945	−9344	−9842	−10,255
283.15	−9585	−9976	−10,435	−10,845
288.15	−10,120	−10,526	−10,990	−11,367
293.15	−10,726	−11,085	−11,507	−11,898
298.15	−11,219	−11,577	−11,994	−12,328
303.15	−11,761	−12,016	−12,439	−12,721
308.15	−12,199	−12,481	−12,874	−13,179
313.15	−12,615	−12,927	−13,292	−13,569

**Table 3:** Values of *∆S*^0^ [J∙mol^−1^·K^−1^] and ∆*H*^0^ [J∙mol^−1^] for ionic liquids 1-hexyl-3-methylimidazolium chloride [HMIM] and 1-methyl-3-octylimidazolium chloride [OMIM] with alkoxy alcohols (2-methoxyethanol, 2-ethoxyethanol, 2-propoxyethanol, 2-butoxyethanol) as a function of temperature.

∆*S*^0^ [J∙mol^−1^·K^−1^],				
1-Hexyl-3-Methylimidazolium Chloride [HMIM]				
T [K]	2-Methoxyethanol	2-Ethoxyethanol	2-Propoxyethanol	2-Butoxyethanol
278.15	113.79	111.08	109.29	107.99
283.15	109.24	106.49	104.56	102.80
288.15	104.69	101.90	99.83	97.61
293.15	100.14	97.31	95.10	92.41
298.15	95.59	92.72	90.37	87.22
303.15	91.04	88.13	85.64	82.03
308.15	86.50	83.54	80.91	76.84
313.15	81.95	78.95	76.18	71.64
1-methyl-3-octylimidazolium chloride [OMIM]				
T [K]	2-methoxyethanol	2-ethoxyethanol	2-propoxyethanol	2-butoxyethanol
278.15	127.38	122.94	119.31	115.41
283.15	121.03	116.78	113.24	109.22
288.15	114.68	110.61	107.17	103.02
293.15	108.34	104.45	101.10	96.83
298.15	101.99	98.29	95.04	90.63
303.15	95.64	92.13	88.97	84.44
308.15	89.29	85.97	82.90	78.24
313.15	82.94	79.80	76.83	72.05
*∆H*^0^ [J∙mol^−1^]				
1-hexyl-3-methylimidazolium chloride [HMIM]				
T [K]	2-methoxyethanol	2-ethoxyethanol	2-propoxyethanol	2-butoxyethanol
278.15	22,226	21,111	20,164	19,476
283.15	20,940	19,801	18,832	17,998
288.15	19,688	18,506	17,477	16,521
293.15	18,323	17,150	16,104	14,981
298.15	17,003	15,803	14,706	13,480
303.15	15,590	14,456	13,297	11,959
308.15	14,225	13,037	11,848	10,325
313.15	12,830	11,587	10,365	8701
1-methyl-3-octylimidazolium chloride [OMIM]				
T [K]	2-methoxyethanol	2-ethoxyethanol	2-propoxyethanol	2-butoxyethanol
278.15	26,486	24,852	23,345	21,847
283.15	24,685	23,089	21,630	20,080
288.15	22,927	21,347	19,892	18,319
293.15	21,033	19,535	18,132	16,487
298.15	19,188	17,728	16,340	14,694
303.15	17,232	15,912	14,531	12,876
308.15	15,317	14,010	12,671	10,931
313.15	13,359	12,064	10,767	8993

**Table 4:** Specification of chemical samples.

Structure	Name, Abbreviation	CAS Number	Purity/%	Source
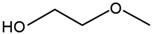	2-Methoxyethanol	109-86-4	≥99	Sigma-Aldrich
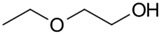	2-Ethoxyethanol	110-80-5	≥99	Sigma–Aldrich
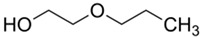	Ethylene glycol monopropyl ether	2807-30-9	≥99	Sigma–Aldrich
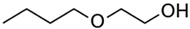	Ethylene glycol butyl ether	111-76-2	≥99	Sigma–Aldrich
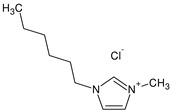	1-Hexyl-3-Methylimidazolium Chloride,	1142-20-7	≥98.5	IoLiTec
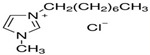	1-Methyl-3-octylimidazolium chloride	64697-40-1	>99.9	IoLiTec

## Data Availability

Data are contained within the article and Supplementary Materials.

## Supplementary Materials

The following supporting information can be downloaded at: https://www.mdpi.com/article/10.3390/molecules28237831/s1, Table S1: The density values [g/cm^3^] for 2-methoxyethanol, 2-ethoxyethanol, 2-propoxyethanol, and 2-butoxyethanol in the temperature range of (278.15–313.15)K at pressure p = 0.1 MPa; Table S2: Molar conductivity values (*Λ*_m_) and concentrations (*m*), for 1-hexyl-3-methylimidazolium chloride [HMIM] with the investigated alkoxy alcohols in the temperature range of (278.15–313.15) K at pressure p = 0.1 MPa ^a^; Table S3: Molar conductivity values (*Λ*_m_) and concentrations (*m*), for 1-methyl-3-octylimidazolium chloride [OMIM] with the investigated alkoxy alcohols in the temperature range (278.15–313.15) K at pressure p = 0.1 MPa ^a^.
