# Supplementary material for: Electrical Conductivity of Ionic Liquids 1-Hexyl-3-Methylimidazolium Chloride (HMIM) and 1-Methyl-3-Octylimidazolium Chloride (OMIM) in Mixtures with Selected Alkoxy Alcohols over a Wide Temperature Range
Source: Molecules. 2023 Nov 28;28(23):7831. doi: 10.3390/molecules28237831 (PMC10707732; doi:10.3390/molecules28237831)
Supplement: Supplementary file 1 [file molecules-28-07831-s001.zip › molecules-2733504-supplementary.pdf]

Electrical Conductivity of Ionic Liquids 1-Hexyl-3-Methylimidazolium Chloride (HMIM) and 1-Methyl-3-Octylimidazolium Chloride (OMIM) in Mixtures with Selected Alkoxy Alcohols Over a Wide Temperature Range.

Zdzisław Kinart,*

University of Lodz, Faculty of Chemistry, Department of Physical Chemistry, 90 – 236 Lodz, Pomorska 163/165, Poland.
Correspondence: zdzislaw.kinart@chemia.uni.lodz.pl

Table S1.

The density values [g/cm^3] for 2-methoxyethanol, 2-ethoxyethanol, 2-propoxyethanol, and 2-butoxyethanol in the temperature range of (278.15 - 313.15)K at pressure p = 0.1 MPa

***T* / K**	***d* [g·cm^{-3}]**	***d* [g·cm^{-3}]**
	**2-methoxyethanol**	**2-ethoxyethanol**
278.15	0.978194	0.943461
283.15	0.973673	0.939013
288.15	0.969132	0.934538
293.15	0.964569	0.930039
298.15	0.959985; 096029 [43];<br>0.602 [44]; 0.960288[45]	0.925515; 0,92515 [43];<br>0.92572 [46]; 0.9258 [47]
303.15	0.955376	0.920960
308.15	0.950740	0.916375
313.15	0.946075	0.911758
	**2-propoxyethanol**	**2-butoxyethanol**
278.15	0.925602	0.912934
283.15	0.921293	0.908817
288.15	0.916960	0.904681
293.15	0.912606	0.900525
298.15	0.908225; 0.80819 [43];<br>0.908233 [48]; 0.9080 [49]	0.896348; 0.89629 [43];<br>0.89625 [50]; 0.8958 [51]
303.15	0.903813	0.892143
308.15	0.899372	0.887914
313.15	0.89490	0.883655

Table S2.

Molar conductivity values (Λ_m) and concentrations (*m*), for 1-hexyl-3-methylimidazolium chloride [HMIM] with the investigated alkoxy alcohols in the temperature range of (278.15 - 313.15)K at pressure p = 0.1 MPa.[a]

10^3 *m* / mol·kg^{-1}	Λ_m / S·cm^2·mol^{-1}							
**1-hexyl-3-methylimidazolium chloride [HMIM] + 2-methoxyethanol**								
T / K	278.15	283.15	288.15	293.15	298.15	303.15	308.15	313.15
0.23745	18.055	20.280	23.168	25.111	27.197	29.438	32.270	34.767
0.56643	18.033	20.254	23.123	25.069	27.128	29.378	32.204	34.644
0.98884	18.019	20.225	23.088	25.04	27.077	29.312	32.121	34.552
2.22058	17.989	20.201	23.019	25.002	26.985	29.22	32.015	34.403
5.79830	17.940	20.130	22.922	24.858	26.809	28.989	31.76	34.052
7.93002	17.918	20.097	22.86	24.792	26.710	28.881	31.598	33.867
9.90124	17.897	20.075	22.831	24.76	26.664	28.814	31.526	33.782
12.6034	17.874	20.047	22.796	24.709	26.601	28.722	31.407	33.658
13.2076	17.869	20.038	22.772	24.695	26.568	28.698	31.387	33.603
14.0645	17.862	20.029	22.758	24.679	26.545	28.671	31.354	33.561
**1-hexyl-3-methylimidazolium chloride [HMIM] + 2-ethoxyethanol**								
0.24438	13.382	15.077	16.909	18.302	20.326	22.250	24.236	26.295
0.60509	13.358	15.046	16.836	18.239	20.267	22.150	24.139	26.156
1.12650	13.334	15.006	16.797	18.195	20.195	22.081	24.025	26.023
2.18114	13.299	14.955	16.723	18.118	20.092	21.964	23.856	25.810
4.22960	13.251	14.905	16.618	17.982	19.955	21.812	23.659	25.566
7.58295	13.190	14.824	16.497	17.849	19.803	21.604	23.403	25.249
9.93656	13.160	14.793	16.420	17.753	19.690	21.515	23.256	25.063
12.6343	13.130	14.754	16.345	17.644	19.584	21.422	23.112	24.882
13.1663	13.121	14.736	16.334	17.625	19.575	21.386	23.081	24.844
14.0265	13.111	14.723	16.313	17.596	19.551	21.354	23.038	24.790
**1-hexyl-3-methylimidazolium chloride [HMIM] + 2-propoxyethanol**								
0.30258	7.011	7.905	8.787	9.396	10.196	11.486	12.261	13.085
0.70278	6.969	7.866	8.715	9.348	10.094	11.372	12.185	12.894
1.25200	6.947	7.834	8.682	9.283	10.022	11.287	12.078	12.748

2.45433	6.897	7.781	8.618	9.179	9.882	11.138	11.872	12.478
5.23500	6.837	7.696	8.515	9.064	9.709	10.910	11.543	12.106
8.69656	6.773	7.608	8.420	8.926	9.516	10.675	11.290	11.739
11.3450	6.735	7.568	8.361	8.861	9.420	10.561	11.049	11.513
14.4187	6.698	7.515	8.299	8.797	9.324	10.419	10.844	11.289
14.9120	6.688	7.510	8.301	8.769	9.288	10.401	10.809	11.243
16.1235	6.674	7.492	8.260	8.740	9.247	10.351	10.739	11.158
**1-hexyl-3-methylimidazolium chloride [HMIM] + 2-butoxyethanol**								
0.19399	3.684	4.011	4.315	4.971	5.237	5.636	6.117	6.585
0.58973	3.663	3.984	4.270	4.937	5.149	5.545	6.021	6.423
1.20753	3.643	3.959	4.229	4.881	5.079	5.452	5.902	6.291
2.26873	3.618	3.929	4.175	4.816	4.963	5.316	5.760	6.104
4.08929	3.588	3.886	4.106	4.758	4.888	5.213	5.605	5.909
7.62441	3.541	3.827	4.021	4.641	4.727	5.022	5.344	5.588
8.40945	3.535	3.816	3.997	4.640	4.707	4.986	5.323	5.546
13.0516	3.494	3.760	3.921	4.560	4.577	4.812	5.113	5.269
13.5770	3.487	3.754	3.913	4.535	4.545	4.783	5.070	5.221
14.3573	3.481	3.746	3.896	4.521	4.523	4.756	5.037	5.178

[a] Standard uncertainties are $u(T)$ = 0.01 K, $u(p)$ = 0.05 MPa, $u(c) = 10^{-4}\cdot c$, and the combined expanded uncertainty is $U_c(\Lambda) = 0.0005\cdot\Lambda$ (level of confidence = 0.95).

Table S3.

Molar conductivity values (Λ_m) and concentrations (m), for 1-methyl-3-octylimidazolium chloride [OMIM] with the investigated alkoxy alcohols in the temperature range (278.15 - 313.15) K at pressure p = 0.1 MPa.[a]

$10^3\ m$ / mol·kg^{-1}	Λ_m / S·cm^2·mol^{-1}							
**1-methyl-3-octylimidazolium chloride [OMIM] + 2-methoxyethanol**								
T / K	278.15	283.15	288.15	293.15	298.15	303.15	308.15	313.15
0.23745	17.355	19.280	22.168	23.910	26.197	28.438	31.270	33.767
0.56643	17.333	19.254	22.123	23.869	26.128	28.378	31.204	33.644
0.98884	17.319	19.225	22.088	23.840	26.077	28.312	31.121	33.552
2.22058	17.289	19.201	22.019	23.802	25.985	28.220	31.015	33.403
5.79830	17.240	19.130	21.922	23.658	25.809	27.989	30.760	33.052

7.93002	17.218	19.097	21.860	23.592	25.710	27.881	30.598	32.867
9.90124	17.197	19.075	21.831	23.560	25.664	27.814	30.526	32.782
12.6034	17.174	19.047	21.796	23.509	25.601	27.722	30.407	32.658
13.2076	17.169	19.038	21.772	23.495	25.568	27.698	30.387	32.603
14.0645	17.162	19.029	21.758	23.479	25.545	27.671	30.354	32.561
**1-methyl-3-octylimidazolium chloride [OMIM] + 2-ethoxyethanol**								
0.24438	12.182	13.577	15.409	17.302	19.026	21.050	22.936	25.195
0.60509	12.158	13.546	15.336	17.239	18.967	20.950	22.839	25.056
1.12650	12.134	13.506	15.297	17.195	18.895	20.881	22.725	24.923
2.18114	12.099	13.455	15.223	17.118	18.792	20.764	22.556	24.710
4.22960	12.051	13.405	15.118	16.982	18.655	20.612	22.359	24.466
7.58295	11.990	13.324	14.997	16.849	18.503	20.404	22.103	24.149
9.93656	11.960	13.293	14.920	16.753	18.390	20.315	21.956	23.963
12.6343	11.930	13.254	14.845	16.644	18.284	20.222	21.812	23.782
13.1663	11.921	13.236	14.834	16.625	18.275	20.186	21.781	23.744
14.0265	11.911	13.223	14.813	16.596	18.251	20.154	21.738	23.690
**1-methyl-3-octylimidazolium chloride [OMIM] + 2-propoxyethanol**								
0.30258	5.811	6.505	7.487	8.296	9.196	10.086	10.961	11.885
0.70278	5.769	6.466	7.415	8.248	9.094	9.972	10.885	11.694
1.25200	5.747	6.434	7.382	8.183	9.022	9.887	10.778	11.548
2.45433	5.697	6.381	7.318	8.079	8.882	9.738	10.572	11.278
5.23500	5.637	6.296	7.215	7.964	8.709	9.510	10.243	10.906
8.69656	5.573	6.208	7.120	7.826	8.516	9.275	9.990	10.539
11.3450	5.535	6.168	7.061	7.761	8.420	9.161	9.749	10.313
14.4187	5.498	6.115	6.999	7.697	8.324	9.019	9.544	10.089
14.9120	5.488	6.110	7.001	7.669	8.288	9.001	9.509	10.043
16.1235	5.474	6.092	6.960	7.640	8.247	8.951	9.439	9.958
**1-methyl-3-octylimidazolium chloride [OMIM] + 2-butoxyethanol**								
0.19399	2.684	2.911	3.115	3.671	3.937	4.436	4.817	5.285
0.58973	2.663	2.884	3.070	3.637	3.849	4.345	4.721	5.123
1.20753	2.643	2.859	3.029	3.581	3.779	4.252	4.602	4.991
2.26873	2.618	2.829	2.975	3.516	3.663	4.116	4.460	4.804

4.08929	2.588	2.786	2.906	3.458	3.588	4.013	4.305	4.609
7.62441	2.541	2.727	2.821	3.341	3.427	3.822	4.044	4.288
8.40945	2.535	2.716	2.797	3.340	3.407	3.786	4.023	4.246
13.0516	2.494	2.660	2.721	3.260	3.277	3.612	3.813	3.969
13.5770	2.487	2.654	2.713	3.235	3.245	3.583	3.770	3.921
14.3573	2.481	2.646	2.696	3.221	3.223	3.556	3.737	3.878

[a] Standard uncertainties are $u(T)$ = 0.01 K, $u(p)$ = 0.05 MPa, $u(c) = 10^{-4} \cdot c$, and the combined expanded uncertainty is $U_c(\Lambda) = 0.0005 \cdot \Lambda$ (level of confidence = 0.95).
